# Black parents’ views and understanding of prenatal genetic testing: a cross-sectional survey of attitudes, knowledge and trust in UK healthcare

**DOI:** 10.1038/s41431-026-02059-0

**Published:** 2026-02-28

**Authors:** Michelle Peter, Clotilde Abe, Agnes Agyepong, Atinuke Awe, Rachael Buabeng, Melissa Dean, Jane Fisher, Sasha Henriques, Kerry Leeson-Beevers, Carol Nelson, Shermel Walters-Lawrence, Lyn S. Chitty, Melissa Hill

**Affiliations:** 1 North Thames Genomic Laboratory Hub, Great Ormond Street Hospital for Children NHS Foundation Trust, London, UK; 2 Genetics and Genomic Medicine, UCL Great Ormond Street Institute of Child Health, London, UK; 3 Five x More, London, UK; 4 Global Child and Maternal Health, London, UK; 5 Mummy’s Day Out, London, UK; 6 Patient and Public Involvement Group Member, London, UK; 7 Antenatal Results and Choices, London, UK; 8 Wellcome Connecting Science, Cambridge, UK; 9 Alström Syndrome UK, Torquay, UK

**Keywords:** Human behaviour, Patient education, Genetic testing, Genetic counselling

## Abstract

Black women in the UK experience disproportionately poor maternal outcomes yet remain underrepresented in research on prenatal screening and diagnostic genetic testing (prenatal testing). We therefore know little about how Black parents feel and what they understand about these tests. Using a cross-sectional online survey, we assessed attitudes towards prenatal tests, knowledge of genetic terms and prenatal tests, and mistrust amongst Black and mixed Black heritage parents in the UK who had been pregnant in the last five years. 110 parents completed the survey (95% female). Screening was valued by most (89%), although only half (50%) reported willingness to undergo invasive diagnostic testing. Preparing for a child with a genetic condition or disability were key motivators for testing, whilst opposition to termination and concerns about miscarriage risk drove refusal. Healthcare professionals (HCPs) were the main source of information when discussing prenatal testing, though mistrust in healthcare systems was high and associated with lower reported uptake of both screening and diagnostic tests. Nearly three-quarters valued speaking to an HCP who shared their ethnic background. Misconceptions about sickle cell were common, with 40% believing it affects only African and Caribbean populations. While most parents recognised the term ‘DNA’, only 28% understood the term ‘genome’. Our findings highlight support for prenatal testing but reveal knowledge gaps and high mistrust that may undermine informed choice. Addressing misconceptions - particularly around sickle cell and available prenatal tests - alongside culturally responsive counselling and community-based education is essential to achieving equitable prenatal care for Black parents.

## Introduction

Prenatal screening and diagnostic genetic tests (prenatal testing) for a range of genetic and chromosomal conditions are integral components of maternity care, allowing parents to manage pregnancy decisions, plan for neonatal care and prepare for their baby’s potential health needs. In England, prenatal testing is delivered through the National Health Service Fetal Anomaly Screening Programme (NHS FASP) [1] – a population screening programme, which offers all pregnant women blood tests and ultrasound examinations to estimate the likelihood of genetic and chromosomal conditions. This includes universally offered screening for thalassaemia, common aneuploidies, and fetal anomalies, as well as targeted screening for sickle cell. If results indicate an increased chance of a condition, diagnostic testing can provide definitive information, though these procedures carry both clinical [2] and psychological impacts [3, 4]. Thus, while early detection offers benefits, a positive or uncertain result can also cause anxiety and decisional conflict [5, 6]. High-quality counselling is, therefore, essential to the ethical delivery of prenatal testing. Midwives typically lead initial discussions about prenatal screening and consent, whilst fetal medicine specialists, clinical geneticists, and genetic counsellors may become involved if an increased chance result or anomaly is identified. Although testing pathways and guidance are nationally specified, there are differences in local workforce capacity and access to specialist expertise [7], meaning that the content and delivery of prenatal testing discussions, and the support available to parents, may vary between local services.

In this context, ensuring equitable prenatal counselling and support is urgent, as racial inequalities mean Black women consistently face disproportionately poor maternal outcomes. Three times more likely than White women to die during pregnancy or shortly after [8], Black women are also at increased risk of stillbirth [9], neonatal death [10], and severe maternal morbidity [11]. While often linked to socioeconomic disadvantage [12, 13], structural racism plays a central role in reinforcing these disparities [14, 15], with Black women reporting racially discriminatory behaviour from healthcare professionals (HCPs) during pregnancy, which shapes their perception of care [16–18] and engagement with maternity services [19]. These experiences, alongside wider social determinants [20, 21], highlight the pressing need to address inequities in maternity care.

Despite their disproportionate risk, little is known about Black parents’ attitudes and knowledge of prenatal testing. Our recent review of 76 UK and US studies on perspectives of prenatal testing found Black participants were rarely included at levels proportionate to national birthing populations [22]. The result is a critical evidence gap in how Black parents navigate the range of testing options offered in routine maternity care. This issue is particularly important in the UK, where prenatal testing is universally offered within a publicly funded healthcare system, and inequities in knowledge or uptake cannot be explained simply by differences in financial access.

The limited research that has focused on Black parents suggests that attitudes towards prenatal testing are shaped by a complex interplay of personal experience, cultural values, and trust [23]. Evidence also indicates that Black parents may experience higher levels of distress following a fetal anomaly diagnosis compared with parents from other ethnic backgrounds [24], further underscoring the importance of culturally responsive counselling and tailored support.

Beyond decision-making and emotional responses, understanding of prenatal tests amongst Black parents remains a crucial but underexplored aspect. To our knowledge, no studies have examined what UK Black parents know about the tests offered through the NHS, or how this shapes their decisions. This gap is particularly concerning given evidence that Black women in the UK are more likely to access antenatal care later in pregnancy [25] and receive fewer checks and scans than women from other ethnic groups [26]. As genomic medicine expands within routine care, this lack of evidence risks further widening health inequalities.

To address this issue, this study used a cross-sectional survey to explore Black parents’ attitudes towards, and knowledge of, prenatal testing in the UK. This study forms part of a larger project that also includes qualitative interviews with Black women about their prenatal testing experiences. Together, these approaches aim to drive the development of equitable services responsive to the communities most at risk.

## Subjects and methods

### Study design

This cross-sectional online survey examined attitudes towards and knowledge of prenatal testing amongst Black parents living in the UK.

### Participants and recruitment

Participants could take part if they: (i) self-reported (using UK census categories) as Black, Black African, Black Caribbean, Black British, or mixed Black heritage; (ii) were aged over 18 years; and (iii) had been pregnant within the UK in the past five years. The survey was hosted on the survey platform REDCap and ran from 15th March 2025 to 15th August 2025. Recruitment used purposive convenience sampling via a cascade approach. Parent and pregnancy organisations, alongside Black-led community groups targeted towards Black parents, advertised the study and shared the link to the survey through their online communities and social media networks. Participation was voluntary. An information sheet preceded the survey, and participants explicitly provided consent to take part.

Ethnicity was self-reported using UK census categories. We use the descriptor “Black” throughout this work, though we acknowledge that this label does not capture the cultural and experiential diversity within Black communities.

### Survey development and patient/public involvement

Survey development was guided by the study’s Patient and Public Involvement Advisory Group (PPIAG) comprising a genetic counsellor, representatives from parent support organisations (including Black-led groups), and Black parents with lived experience of pregnancy and prenatal testing. MP, an experienced researcher in maternal health, prenatal genomics, and survey design, developed the survey from a literature review and findings from in-depth interviews with 39 Black mothers offered prenatal testing [27]. PPIAG members reviewed draft questions for clarity and cultural sensitivity, suggesting refinements to reflect the experiences of Black parents in maternity care. A midwife known to the first author further verified knowledge-based items for accuracy with NHS prenatal testing guidelines. The survey was piloted with 10 people (not included in the final analysis) and feedback was incorporated prior to survey launch.

The final survey (Supplementary information) included closed-response items addressing: 1) Attitudes towards and willingness to undergo prenatal testing; 2) Factors influencing decision-making around prenatal testing; 3) Medical mistrust; 4) Perceived and objective knowledge of prenatal tests; 5) Demographic characteristics (optional for all participants).

### Measures

#### Attitudes towards prenatal testing

Participants read a description of prenatal screening and invasive diagnostic testing before being asked about their attitudes towards these tests. Attitudes were measured with four Likert-scale items (1 = strongly disagree, 5 = strongly agree), adapted from previous research [28], and summed to create total attitude scores.

#### Medical mistrust

The Group-based Medical Mistrust Scale (GBMMS) [29], a validated 12-item measure, assessed perceptions of race-based inequities in healthcare. The term ‘healthcare professional’ replaced the term ‘doctor’ to reflect UK maternity care, which includes midwives and doctors. Items are rated on a 5-point Likert scale (1 = strongly disagree to 5 = strongly agree); higher scores indicate greater medical mistrust. The scale produces a total score and three subscale scores: Suspicion, Group-based Disparities, and Lack of Support from Healthcare Professionals.

#### Knowledge of prenatal testing

Items examining perceived knowledge of genetic terminology were informed by previous research [30] and assessed familiarity with and understanding of four words: ‘DNA’, ‘chromosome’, ‘gene’, and ‘genome’. Multiple-choice questions, developed by MP, assessed objective knowledge of prenatal tests, and were scored correct/incorrect. Each set of objective knowledge questions was preceded by a short description of the relevant test and how it is offered within the NHS, followed by a brief hypothetical scenario.

### Data analysis

Descriptive and inferential statistics were reported. Internal consistency of the GBMMS was assessed using Cronbach’s alpha. One-way analysis of variance (ANOVA) and Kruskal–Wallis tests assessed group differences, and post-hoc pairwise tests were conducted when omnibus tests were significant. Spearman’s rank-order correlations assessed associations between ordinal or non-normal variables. Effect sizes were reported as eta squared (η²) for ANOVA and correlation coefficients (ρ) for non-parametric associations. Statistical significance was set at *p* < 0.05. Analyses were performed in R (version 4.4.1) [31]. Only complete responses for each survey question were included in the analyses. As this was an exploratory study, no a priori sample size calculation was undertaken.

## Results

### Participant characteristics

A total of 110 Black parents participated in the survey. Of those who provided demographic information, around half (49.5%; *n* = 51) were aged 35–44 years. Most were female (95.1%; *n* = 97), identified as Christian (89.5%; *n* = 68), and 47.5% (*n* = 48) were educated to undergraduate level. Few reported a genetic condition themselves (4%; *n* = 4), and only 10.8% (*n* = 11) had a child who has or is a carrier of a genetic condition (Table 1).

**Table 1:** Participant characteristics.

	*N* (%)		*N* (%)
Gender		Do you have a genetic condition?	
Female	97 (95.1)	Yes	4 (4)
Male	5 (4.9)	No	96 (95)
**Age, years**		Don’t know	1 (1)
25–34	47 (45.6)	**Are you a carrier of a genetic condition?**	
35–44	51 (49.5)	Yes	13 (12.9)
45–54	5 (4.9)	No	82 (81.2)
**Ethnicity**		Don’t know	6 (5.9)
Black/Black British/Black African	40 (39.6)	**Do you have a child who has/ is a carrier of a genetic condition?**	
Black/Black British/Black Caribbean	31 (30.7)		
Mixed Black heritage	25 (24.2)	Yes	11 (10.8)
Black African and Black Caribbean	5 (5)	No	78 (76.5)
**Education**		Don’t know	11 (10.8)
Undergraduate	48 (47.5)	Not applicable	2 (2)
Postgraduate	43 (42.6)		
Secondary education	5 (5)		
Vocational	5 (5)		
**Religiosity**			
Very	33 (32.4)		
Somewhat	44 (43.1)		
Not at all	23 (22.5)		
**Religion**			
Christian	68 (89.5)		
Muslim	2 (2.6)		
None	6 (7.9)		

Categories do not reflect the total number of survey participants since provision of this information was optional; percentages are calculated over known information.

### Attitudes towards and decision-making around prenatal testing

#### General attitudes towards prenatal testing

Participants rated the extent to which they felt prenatal screening and invasive diagnostic testing was beneficial, a good thing, helpful, and important (1 = strongly disagree, 5 = strongly agree) (Table 1; Supplementary information).

##### Prenatal screening

Attitudes were highly positive; the mean total score was 18.4 (*SD* = 2.93; 95% CI [17.87, 18.98]) (out of a possible 20). There was a significant effect of ethnicity, *F*(3, 97) = 4.96, *p* = 0.003, *η*² = 0.13, with Black Caribbean participants scoring lower (*M* = 16.9) than Black African participants (*M* = 19.4; *p* = 0.002). No significant effects for age, education or religiosity were found.

##### Diagnostic testing

Attitudes were positive but more variable, with a mean total score of 15.1 (*SD* = 4.02; 95% CI [14.38, 15.90]). No differences were observed by ethnicity, age, education, or religiosity.

### Willingness to have prenatal testing

Participants were asked if they would have prenatal screening and diagnostic testing. Whilst most said they would have screening (89.1%; *n* = 98), only half were willing to have a diagnostic test (50%; *n* = 55). Those willing to have screening had more positive attitudes towards screening [*F*(2, 107) = 48.36, *p* < 0.001, η² = 0.48], with attitude scores highest amongst those who indicated they would have screening (*M* = 19.1, *SD* = 1.86; 95% CI [18.70, 19.50]), compared with those who said they were unsure (*M* = 16.2, *SD* = 3.86; 95% CI [10.10, 22.40]) and those who would not have screening (*M* = 11.5, *SD* = 4.00; 95% CI [8.16, 14.80]). Similarly, attitudes towards diagnostic testing were more positive amongst those willing to have diagnostic testing [*F*(2, 107) = 39.66, *p* < 0.001, η² = 0.43]. Attitude scores were highest amongst those who reported that they would have diagnostic testing (*M* = 17.5, *SD* = 2.86; 95% CI [16.70, 18.30]), compared with those who were unsure (*M* = 14.4, *SD* = 3.11; 95% CI [13.20, 15.60]) or who would not have diagnostic testing (*M* = 11.2, *SD* = 3.46; 95% CI [9.78, 12.50]).Thus, willingness to undergo prenatal testing was linked to more positive attitudes, with screening widely accepted but greater reservations about diagnostic testing.

### Factors influencing the decision to have prenatal testing

Those willing to undergo prenatal screening and diagnostic testing could select up to two reasons that best reflected their decision. The main motivations for those willing to undergo these tests were practical, most notably preparing for a child with a genetic condition (screening: 59.2%; *n* = 58; diagnostic testing: 47.3%; *n* = 26) and preparing for a child with a disability (screening: 46.9%; *n* = 46, diagnostic testing: 36.4%; *n* = 20) (Tables 2 and 3, Supplementary information). Social influences were rarely endorsed.

**Table 2:** Descriptive statistics for total and subscale scores on the Group-Based Medical Mistrust Scale (GBMMS).

	*N*	Mean	SD	95% CI	Median	IQR	Range
Total GBMMS scale (possible range 12–60)	105	38.23	6.8	36.83–39.50	39	7	20–50
Subscales:							
Lack of support from HCPs (possible range 3–15)	105	10.04	2.19	9.61–10.47	10	3	5–14
Suspicion (possible range 6–30)	105	17.1	4.32	16.21–17.91	18	5	7–26
Group-based health disparities (possible range 3–15)	105	11.09	1.71	10.73–11.4	12	1	3–12
HCPs sometimes hide information from patients who belong to my ethnic group.	105	3.42	1.01	3.22–3.61	3	1	1–5
HCPs have the best interests of people of my ethnic group in mind^a^.	105	3.36	0.79	3.21–3.51	4	1	1–4
People of my ethnic group should not confide in HCPs because it will be used against them.	105	2.52	0.98	2.33–2.71	2	1	1–5
People of my ethnic group should be suspicious of information from HCPs.	105	2.7	0.95	2.51–2.88	3	1	1–5
People of my ethnic group cannot trust HCPs.	105	2.68	1.06	2.47–2.88	3	1	0–5
People of my ethnic group should be suspicious of modern medicine.	105	2.4	1.05	2.2–2.6	2	1	0–5
HCPs treat people of my ethnic group like ‘guinea pigs’.	105	2.82	1.07	2.61–3.03	3	1	0–5
People of my ethnic group receive the same medical care from HCPs as people from other groups^a^.	105	3.66	0.78	3.51–3.81	4	0	0–4
HCPs do not take the medical complaints of people of my ethnic group seriously.	105	3.99	0.99	3.8–4.18	4	1	0–5
People of my ethnic group are treated the same as people of other groups by HCPs^a^.	105	3.76	0.6	3.65–3.88	4	0	1–4
In most hospitals, people from all ethnic groups receive the same kind of care^a^.	105	3.67	0.73	3.53–3.81	4	0	0–4
I have personally been poorly or unfairly by HCPs because of my ethnicity.	105	3.26	1.27	3.01–3.5	3	2	1–5

*SD* standard deviation, *CI* confidence interval, *IQR* interquartile range.

^a^Item is positively worded and is reverse scored.

**Table 3:** Descriptive statistics for the total knowledge score and subscales of different prenatal tests.

	*N*	Mean	SD	95% CI	Median	IQR	Range
Overall knowledge score	106	14.42	4.77	13.50–15.33	15	6	0–23
Combined screening test	106	4.09	1.15	3.87–4.32	4	1	0–5
Quadruple test	106	1.40	1.13	1.18–1.61	1	2	0–4
NIPT	106	2.50	1.66	2.18–2.82	3	3	0–5
Amniocentesis	106	1.37	1.08	1.16–1.58	1	2	0–3
Sickle cell - part 1	106	3.01	1.03	2.81–3.21	3	1	0–4
Sickle cell - part 2	106	2.05	1.05	1.84–2.25	2	1	0–3

*SD* standard deviation, *CI* confidence interval, *IQR* interquartile range, *NIPT* Non-invasive prenatal testing.

Those who would not undergo screening or diagnostic testing could select up to two reasons for their decision. Opposition to pregnancy termination was the overwhelming reason amongst those who would decline screening, (100%; *n* = 8). For diagnostic testing, concern about miscarriage risk dominated (85.2%; *n* = 23), with fewer citing reasons such as procedural pain (3.7%; *n* = 1) and partner opposition (0%; *n* = 0).

**Fig. 1:**
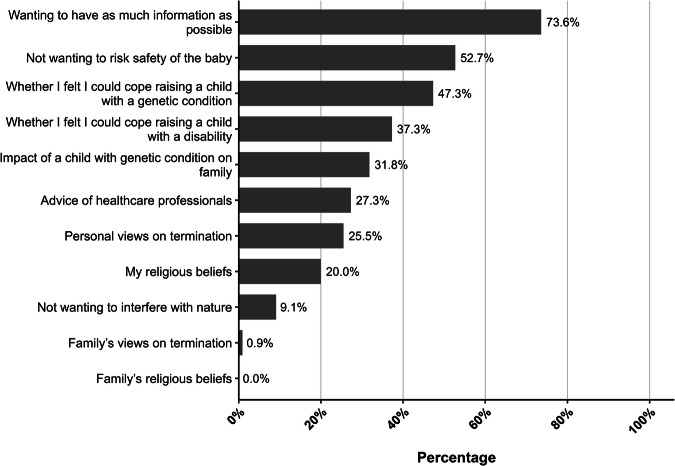
Factors infl uencing decisions about prenatal testing. Percentage of respondents selecting each factor that would infl uence their decision about whether toundergo prenatal testing. Participants were permitted to select up to four response options.

Participants were also asked which factors would influence their decision about prenatal testing. The main considerations were wanting as much information as possible (73.6%; *n* = 81), not risking the baby’s safety (52.7%; *n* = 58), and whether they felt able to cope with raising a child with a genetic condition (47.3%; *n* = 52) (Fig. 1).

### Sources of information accessed when deciding on prenatal testing

When making decisions about prenatal testing, HCPs were the most frequently reported source of information (88.2%; *n* = 97), followed by the Internet (64.5%; *n* = 71). In contrast, religious leaders were rarely selected (6.4%; *n* = 7) (Table 4; Supplementary information).

**Table 4:** Percentage of correct responses for objective knowledge items.

	*N* (%)
Combined screening test (CST)	
The CST tests the chance of a baby having Down syndrome, Edwards’ syndrome and Patau’s syndrome.	79 (76)
A low chance result from CST in a previous pregnancy means the result will be the same in a current pregnancy	92 (87.6)
The CST tests for autism.	92 (88.5)
The CST cannot harm the mother or the baby.	70 (66.7)
Down syndrome only happens in pregnancies of women over 35.	99 (94.3)
**Quadruple test (QT)**	
The QT is less accurate than the CST.	17 (16.3)
The QT tests the chance of a baby having Down syndrome, Edwards’ syndrome and Patau’s syndrome.	16 (15.2)
A high chance result from a QT means that a baby has a genetic condition for certain.	62 (59)
You can find out the baby’s sex from the QT.	53 (50.5)
**Non-invasive prenatal testing (NIPT)**	
NIPT comes with a risk of complications for the mother and the baby.	51 (49.5)
NIPT is offered in all pregnancies.	69 (69)
NIPT will tell you for certain whether the baby has Edwards’ syndrome.	47 (45.6)
NIPT screens for all genetic conditions.	51 (49.5)
If you receive a high chance result from NIPT, you will need further invasive testing to find out for certain if the baby has Edwards’ syndrome.	47 (46.1)
**Amniocentesis**	
Amniocentesis will tell you for certain if the baby has Patau’s syndrome.	43 (42.2)
Amniocentesis is completely safe to the mother and the baby.	68 (66.7)
Amniocentesis can be performed as early as 10 weeks in pregnancy.	34 (33)
**Sickle cell - part 1**	
Sickle cell is a genetic condition and is not contagious.	99 (96.1)
A baby cannot be affected by sickle cell if both parents do not have sickle cell but are only carriers of the condition.	89 (87.3)
A baby cannot be affected by sickle cell because children from two previous pregnancies do not have sickle cell.	94 (93.1)
The chance that a baby, whose parents are both sickle cell carriers, will be affected by sickle cell is: zero chance; 1 in 4 (25%); 1 in 2 (50%); 1 in 1 (100%)	37 (35.9)
**Sickle cell - part 2**	
Sickle cell only affects people of African and Caribbean descent.	62 (60.2)
If one parent has sickle cell trait but the other does not, the baby will not have sickle cell or the sickle cell trait.	70 (68)
Sickle cell trait will eventually develop into sickle cell.	85 (82.5)

Items are paraphrased for clarity in this table; complete item wording as presented to participants is available in the survey.

When asked whether it would be important to speak to someone from the same ethnic group when deciding on prenatal testing, nearly three-quarters considered this to be important or very important (73.4%; *n* = 80). Participants were also asked about their awareness of UK support organisations for parents during pregnancy. Recognition was highest for Five x More, an organisation providing pregnancy and birth resources for Black parents (66.4%; *n* = 73) (Table 5; Supplementary information).

### Trust in healthcare and attitudes towards prenatal testing

#### The Group-Based Medical Mistrust Scale (GBMMS)

The GBMMS was used to assess mistrust in healthcare. The total GBMMS scale showed strong internal consistency (α = 0.83), supporting use of the total score as a reliable measure of medical mistrust. Internal consistency of subscales was mixed: Suspicion was good (α = 0.80), Group-based Disparities was moderate (α = 0.73), and Lack of Support was weak (α = 0.49). For the total GBMMS scale, participants scored a mean of 38.2 (*SD* = 6.8, 95% CI [36.83, 39.5]) out of a possible 60 (Table 2), reflecting moderate to high levels of mistrust. At the subscale level, Suspicion had the highest mean (M = 17.1, *SD* = 4.32, 95% CI [16.21, 17.91]). Notably, agreement was strongest for statements reflecting perceived disparities and lack of fairness.

**Fig. 2:**
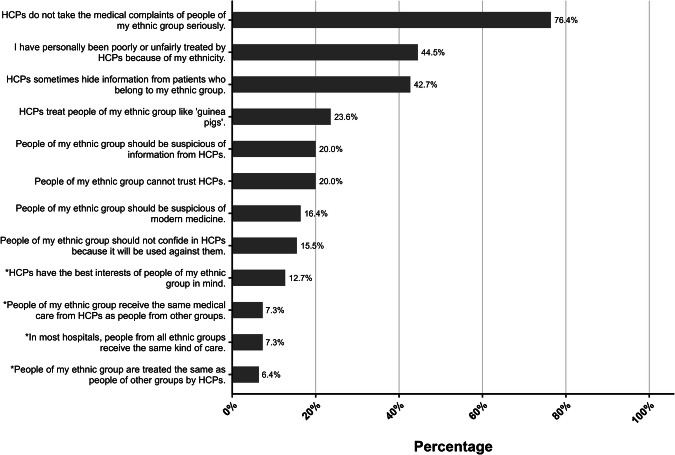
Agreement with items on the Group-Based Medical Mistrust Scale (GBMMS). Percentage of participants who agreed or strongly agreed with each GBMMS item. Participants ratedtheir personal views towards the healthcare system on a Likert scale ranging from strongly agree to strongly disagree.Items marked with an asterisk are positively worded and are reverse scored.

The most widely endorsed statement was that “*Healthcare professionals do not take the medical complaints of people of my ethnic group seriously*” (76.4%; *n* = 84) (Fig. 2). Nearly half reported that they had personally been unfairly treated by HCPs because of their ethnicity (44.5%; *n* = 49), and only 13.1% agreed that HCPs have the best interests of their ethnic group in mind (12.7%; *n* = 14). Fewer than 10% felt that people of their ethnic group receive the same medical care as those from other ethnic groups (7.3%; *n* = 8).

Total GBMMS scores were negatively correlated with both attitudes towards screening (ρ = –0.29, *p* = 0.003) and diagnostic testing (ρ = –0.28, *p* = 0.004), indicating that higher mistrust was linked to less positive views of both procedures. Mistrust was also significantly higher (ρ = –0.25, *p* = 0.010) amongst those who rated speaking with someone from their own ethnic group as very important (M = 39.8, *SD* = 6.12) compared to those who viewed this as very unimportant (M = 34.4, *SD* = 7.37). No significant differences in mistrust were observed across age, ethnicity, education, or religiosity.

### Knowledge of genetics and the tests that are offered in pregnancy

#### Perceived knowledge of genetic terminology

Participants were asked whether they had heard of and knew the meaning of four genetic words (Table 5; Supplementary information). All had heard of ‘DNA’ (100%; *n* = 104), whilst familiarity with the word ‘genome’ was lower (79.1%; *n* = 83). Knowledge of the words’ meanings showed a similar pattern, with only 37.1% (*n* = 39) knowing the meaning of ‘genome’. Ethnicity was significantly associated with having heard these terms (χ²(3) = 11.03, *p* = 0.012), with more participants of mixed Black heritage reporting having heard these words. No significant associations were observed for age or education.

#### Objective knowledge of prenatal testing

Participants were assessed on their knowledge of the different genetic tests offered in pregnancy. Overall knowledge was modest: The total knowledge score ranged from 0 to 23 (out of a possible 24), with a mean of 14.4 (*SD* = 4.77, 95% CI [13.5, 15.3]) (Table 3).

Participants’ knowledge was strong for the combined screening test (CST), with 88.5% (*n* = 92) correctly answering that it does not test for autism (Table 4). Knowledge of sickle cell testing was also generally high - although only 35.9% (*n* = 37) correctly identified that if both parents are sickle cell carriers, the chance of having an affected baby is 1 in 4. Knowledge was lowest for NIPT and the quadruple test (QT): just 15.2% (*n* = 16) correctly answered that the QT is less accurate than the CST, and only 16.3% (*n* = 17) recognised that it does not test for Edwards’ and Patau’s syndrome.

No significant differences in objective knowledge were noted across age, education or ethnicity. However, there was a significant positive correlation between total knowledge score and the number of genetic words participants reported having heard of (ρ = 0.24, *p* = 0.013), indicating that those who had heard of more genetic terms had better actual knowledge of prenatal genetic tests.

## Discussion

This study found that Black parents strongly supported prenatal screening, whilst attitudes towards invasive diagnostic testing were more variable. Decisions were shaped by preparation and information needs, alongside opposition to termination and concerns about miscarriage risk. Healthcare professionals (HCPs) were viewed as the primary source of information regarding prenatal testing, though mistrust in healthcare was common and influenced testing uptake. Knowledge of prenatal tests was variable: highest for common tests and terms but weaker for less commonly offered screening tests and genomics.

### Motivations for and attitudes towards prenatal testing

Consistent with previous research, the primary motivations for undergoing prenatal testing amongst Black parents in this study were information gathering and preparation for a child with a genetic condition or disability. Studies in the UK and internationally have similarly found that many parents pursue testing for reassurance and preparation [32–34], underscoring its perceived practical and informational value.

We found that screening was more strongly favoured than diagnostic testing. This could be because parents perceive it to be risk-free. Our own research with Black and South Asian parents showed that screening is often viewed as “a simple blood test” and a first opportunity to see the baby [23] – a perception that may leave parents unprepared for news that their baby may have a genetic condition, for the offer of diagnostic testing and for decisions around termination of pregnancy that can follow an increased-chance result. At the same time, it is notable that, in the current study, opposition to termination emerged as the leading reason for declining screening. This aligns with other work showing that, even when parents value information about their baby’s health, opposition to termination can outweigh the perceived benefits of screening [35, 36].

It is important, therefore, that pre-test counselling outlines the preparatory benefits of testing whilst supporting parental autonomy, ensuring that those opposed to termination understand that they can still access testing to gain information. For Black parents in particular whose decision-making may be further shaped by mistrust of healthcare systems and experiences of discrimination [23], ensuring access to non-directive, culturally sensitive counselling is critical.

### Ethnic concordance and mistrust

Attitudes towards prenatal testing in this study were primarily guided by practical considerations, with family and social influences playing a smaller role. Critically, the finding that nearly three-quarters of Black parents valued speaking to someone from their own ethnic group when making testing decisions (ethnic concordance) highlights the importance of culturally sensitive communication for Black parents in this study. Evidence from wider healthcare research shows that ethnic concordance between patients and professionals can improve communication, trust, and satisfaction, particularly where communities have experienced systemic discrimination [37, 38]. For prenatal testing, culturally concordant counselling may foster a sense of safety and relatability, but it is important to recognise that concordance alone does not ensure high-quality care. Culturally sensitive approaches remain essential to providing supportive and effective counselling, regardless of the HCP’s background.

The value placed on culturally matched care in this study also connects clearly to findings on mistrust. Participants reported high levels of group-based medical mistrust, particularly around unequal treatment, dismissal of symptoms, and hidden information. Higher mistrust was linked to lower uptake of both screening and diagnostic testing, and to greater emphasis on culturally concordant communication. This suggests that cultural concordance may not only enhance comfort for Black parents but also help reduce the impact of mistrust on decisions about prenatal testing.

These findings should be understood in the context of wider evidence on Black people’s experiences of healthcare where historical and ongoing structural racism have contributed to a legacy of mistrust [39]. This mistrust extends into the field of genetics. Black communities have historically been subjected to unethical practices, including non-consensual testing [40], exploitation of biospecimens [41], and exclusion from genomic research [42]. These legacies, combined with ongoing underrepresentation in genetic studies [43], have left many Black communities wary of genetic research and how genetic information might be used against them [44, 45]. The desire for ethnically concordant care and the association between mistrust and lower uptake of testing reflect how these historical and structural inequities continue to shape present-day attitudes and behaviours.

Several approaches could help address these challenges, such as improving training for HCPs in culturally competent communication, co-developing resources with Black parent groups, and ensuring that maternity and prenatal testing services acknowledge past inequities. In practice, this might include integrating this history into staff training, so that concerns about mistrust are recognised and addressed openly.

### Knowledge of prenatal testing and genetics

Overall, knowledge of prenatal tests was moderate. Black parents in this study demonstrated relatively good understanding of the combined screening test (CST) but had notable gaps in their knowledge of the quadruple test (QT). One possibility is that the CST is presented as the “default” screening option, whereas the QT is only offered later in pregnancy if combined screening is not possible. This positioning may mean that fewer parents are familiar with the limitations of the QT. Clear information about test accuracy is essential to support informed choice, and public knowledge of the QT in particular is important given its lesser accuracy [46]. National resources already exist; however, our findings suggest that these may not be reaching all parents effectively. Collaboration with trusted community-led organisations, such as Five X More, which many participants in this study recognised, could strengthen the reach and relevance of information. Embedding NHS-approved resources within such platforms, and ensuring materials are culturally appropriate (for example, using representative images or videos delivered by Black healthcare professionals), may make information more engaging and better aligned with parents’ needs.

Beyond these general knowledge gaps, this study also revealed important misconceptions around sickle cell. While most recognised the condition as genetic and not contagious, many misunderstood its prevalence, and almost 40% believed it only affects people of African or Caribbean ancestry. This reflects a common stereotype that associates sickle cell solely with these populations [47]. Such stereotypes appear to form part of wider racialised beliefs about genetic conditions and inheritance held by some Black parents, particularly around who is affected by certain conditions, as suggested by qualitative work from our research group. These beliefs may be further reinforced by healthcare practices, including NHS screening approaches such as the Family Origin Questionnaire – a checklist that assigns high risk of sickle cell to countries with high prevalence of the condition, many of which also have large Black populations. Given this, and the fact that sickle cell is among the most common genetic conditions in the UK [47], providing accurate information to parents is essential. Evidence shows that education delivered in community settings can effectively increase awareness among pregnant women [48], highlighting the value of targeted approaches. However, these gaps are not limited to parents: poor understanding of sickle cell has also been documented amongst HCPs, with serious consequences for patient safety [49]. Strengthening education for both communities and HCPs is therefore essential.

While most parents were familiar with genetic words like ‘DNA’, few understood the word ‘genome’, reflecting broader evidence that public familiarity with genomics remains limited [50]. Addressing this knowledge gap is especially pressing as genomic medicine becomes embedded in routine maternity service and initiatives like the Generation Study [51] (which consents parents prenatally for newborn sequencing) expand access to genomic technologies. Ensuring that all parents, particularly Black communities, who remain underrepresented in genomics research, are equipped to engage with these developments will be essential.

**Fig. 3:**
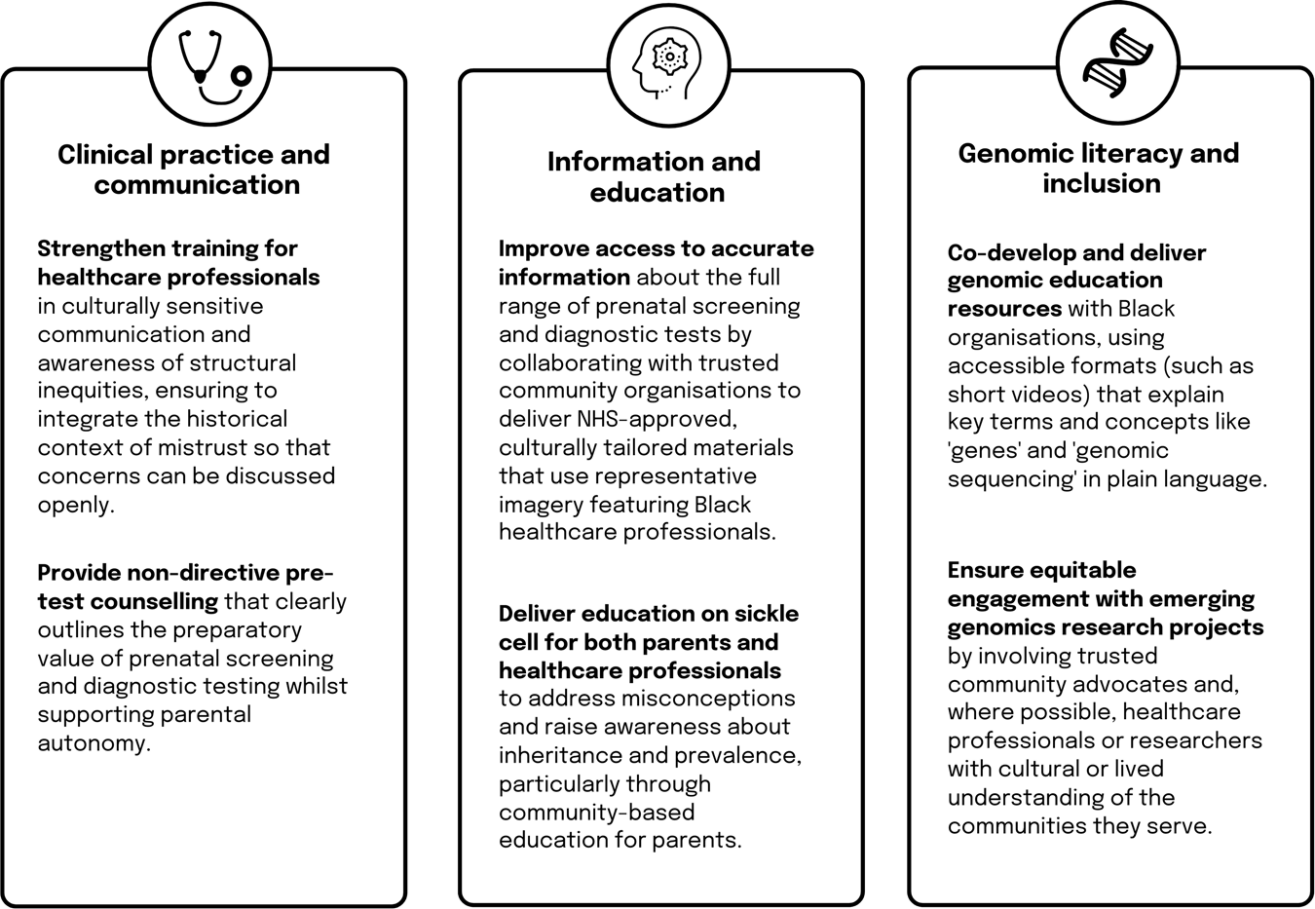
Practice and policy recommendations to support equitable prenatal testing services. Summary of recommended actions developed in response to the study fi ndings, organised acrossthree domains: clinical practice and communication; information and education; and genomic literacy and inclusion.

A summary of key practice and policy recommendations can be viewed in Fig. 3.

### Strengths and limitations

This study has notable strengths, including being the first UK survey to examine Black parents’ attitudes and knowledge of prenatal screening and diagnostic testing. Co-design with a patient and public advisory group enhanced cultural sensitivity, and the survey achieved a large sample for this under-researched population, capturing a wide range of domains across attitudes, knowledge, and trust. However, several limitations should be considered when interpreting the findings. As an exploratory study using purposive convenience sampling, the results should not be interpreted as statistically representative of all Black parents in the UK. The sample was predominantly female, well-educated, and the survey was presented in English, which may limit the generalisability of findings. In addition, the use of closed survey items may have under-captured the role of family, faith, and social networks and limited our ability to explore why particular attitudes, knowledge gaps, or decision-making patterns were observed. Parity and prior adverse pregnancy outcomes were also not assessed and may have influenced parents’ responses. Finally, although the study included parents from different Black backgrounds, relatively small numbers within distinct ethnic subgroups limited exploration of culturally specific perspectives within the wider Black population. Future work should broaden within-group diversity and use qualitative methods to examine cultural influences and engagement with emerging genomic technologies.

## Conclusion

This study shows that, whilst Black parents in the UK are generally supportive of prenatal screening, knowledge gaps and high levels of mistrust shape decision-making. Attitudes were driven by practical and informational needs, yet invasive diagnostic testing was approached with greater caution, and mistrust was linked to lower reported uptake of both screening and diagnostic procedures. Misconceptions around sickle cell inheritance, limited understanding of the quadruple test and NIPT, and low familiarity with genomics highlight the need for preparation for emerging technologies. Addressing these issues will require culturally responsive communication and community-based education delivered in partnership with trusted organisations to support Black parents to engage confidently with the changing landscape of prenatal genetic testing.

Looking ahead, future research should focus on how genomic information is communicated and interpreted in prenatal care. This includes the development and evaluation of models of prenatal genetic counselling and community-level information provision that support engagement with genomic testing among Black parents in the context of existing knowledge gaps and mistrust.

## Supplementary information

Tables and Survey

## Data Availability

The raw data that support the findings of this study are available from the first author upon request.

## Code availability

The R code used to analyse the data are available from the first author upon request.
