# Supplementary material for: Black parents’ views and understanding of prenatal genetic testing: a cross-sectional survey of attitudes, knowledge and trust in UK healthcare
Source: Eur J Hum Genet. 2026 Feb 28;34(7):1005–14. doi: 10.1038/s41431-026-02059-0 (PMC13342292; doi:10.1038/s41431-026-02059-0)
Supplement: Supplementary file 1 — Tables and Survey [file 41431_2026_2059_MOESM1_ESM.pdf]

Supplementary information

The following six tables present descriptive and inferential statistics related to the survey findings. All tables have been cited in the main manuscript.

Table 1. General attitudes towards prenatal testing.

	N	Mean	SD	95% CI	Median	IQR	Range
**For me, screening tests in pregnancy are**							
Harmful (1) - Beneficial (5)	110	4.6	0.78	4.45-4.75	5	0.75	1-5
Unimportant (1) - Important (5)	109	4.64	0.81	4.49-4.80	5	0	1-5
A bad thing (1) - A good thing (5)	108	4.66	0.73	4.52-4.80	5	0	2-5
Unhelpful (1) - Helpful (5)	110	4.65	0.76	4.51-4.80	5	0	2-5
Total score	110	18.43	2.93	17.87-18.98	20	2	7-20
**For me, diagnostic tests in pregnancy are**							
Harmful (1) - Beneficial (5)	110	3.47	1.31	3.22-3.72	3	2	1-5
Unimportant (1) - Important (5)	103	4.04	1.01	3.84-4.24	4	2	1-5
A bad thing (1) - A good thing (5)	109	3.97	0.97	3.79-4.16	4	2	2-5
Unhelpful (1) - Helpful (5)	107	4.06	0.99	3.87-4.25	4	2	1-5
Total score	110	15.14	4.02	14.38-15.9	15	7.5	3-20

Key: SD = standard deviation; CI =confidence interval; IQR = interquartile range

Table 2. Reasons for having and not having prenatal screening.

	N (%)
**Which of these bests describe why you would have a screening test?**	
So I can prepare for the possibility of having a child with a genetic condition.	58 (59.2)
So I can prepare for the possibility of having a child with a disability.	46 (46.9)
To help me make a decision about whether or not to continue with the pregnancy.	29 (29.6)
I would want as much information about the baby as possible.	39 (39.8)
My partner or family would want me to.	2 (2)
My friends have had screening in their pregnancies.	0 (0)
It is part of the antenatal care service.	12 (12.2)
**Which of these bests describe why you would not have a screening test?**	
I would never terminate an affected pregnancy.	8 (100)
It would cause a lot of anxiety if I found out there was a high chance of having a baby with a genetic condition.	1 (12.5)
I wouldn't want to have to make a decision about what to do next.	0 (0)
My partner or family would not want me to.	0 (0)
It would cause a lot of anxiety if I found out there was a high chance of having a baby with a disability.	1 (12.5)
It doesn't give me a certain result.	3 (37.5)
I would prefer not to know.	2 (25)

Note: Participants could select up to two responses, so percentages represent the proportion of participants choosing each option and may total more than 100%.

Table 3. Reasons for having and not having diagnostic testing.

	N (%)
**Which of these bests describe why you would have diagnostic testing?**	
So I can prepare for the possibility of having a child with a genetic condition.	26 (47.3)
So I can prepare for the possibility of having a child with a disability.	20 (36.4)
To help me make a decision about whether or not to continue with the pregnancy.	20 (36.4)
I would want as much information about the baby as possible.	26 (47.3)
My partner or family would want me to.	1 (1.8)
My friends have had diagnostic testing in their pregnancies.	0 (0)
It is part of the antenatal care service.	8 (14.5)
**Which of these bests describe why you would not have diagnostic testing?**	
Because of the risk of miscarriage.	23 (85.2)
Because the procedure might be painful or uncomfortable.	1 (3.7)
I would never terminate an affected pregnancy.	10 (37)
My partner or family would not want me to.	0 (0)
It would cause a lot of anxiety if the baby was found to be affected.	5 (18.5)
I wouldn't want to have to make a decision about whether to terminate the pregnancy.	6 (22.2)
I would prefer not to know.	2 (7.4)

Note: Participants could select up to two responses, so percentages represent the proportion of participants choosing each option and may total more than 100%.

Table 4. Percentage of participants who strongly/agreed with sources of information consulted when making decisions about prenatal testing.

	N (%)
Healthcare professionals	97 (88.2)
The Internet	71 (64.5)
Parent support organisations	65 (59.1)
Friends and family	59 (53.6)
Religious leaders	7 (6.4)

Table 5. Awareness of parent support organisations.

	N (%)
Five x More	73 (66.4)
Tommy's Helpline for Black and Black Mixed-heritage women	53 (48.2)
Antenatal Results and Choices	14 (12.7)
None of these	25 (22.7)

Table 6. Descriptive statistics for total number of genetic terms heard of and understood.

	N	Mean	SD	95% CI	Median	IQR	Range
Total: Genetic words heard of	106	3.75	0.49	3.66-3.85	4	0	2-4
Total: Genetic words understood	106	3.18	0.81	3.02-3.33	3	1	0-4

Key: SD = standard deviation; CI =confidence interval; IQR = interquartile range

Presented overleaf is the survey that participants were asked to complete.

Survey Progress:

Part 1: Views on prenatal testing and attitudes towards the healthcare system

In this section, you'll be asked about your views on the different types of screening and diagnostic tests that are offered in pregnancy.

Please read the following information about screening tests in pregnancy. Some screening tests in pregnancy can help detect the chance of a baby having a genetic condition such as Down syndrome, Edwards' syndrome, or Patau's syndrome. Screening tests tell you if your baby has a low chance (lower than 1 in 150) or a high chance (higher than 1 in 150) of having one of these conditions, but they don't tell you if the baby has the condition for certain. Screening tests come with no risks to the baby and involve taking a sample of the mother's blood (from the arm like a normal blood test) and/or an ultrasound scan. Now please answer the following questions.

For me, screening tests in pregnancy are

◯ 1 - Harmful
◯ 2
◯ 3
◯ 4
◯ 5 - Beneficial

For me, screening tests in pregnancy are

◯ 1 - Unimportant
◯ 2
◯ 3
◯ 4
◯ 5 - Important

For me, screening tests in pregnancy are

◯ 1 - A bad thing
◯ 2
◯ 3
◯ 4
◯ 5 - A good thing

For me, screening tests in pregnancy are

◯ 1 - Unhelpful
◯ 2
◯ 3
◯ 4
◯ 5 - Helpful

Would you have a screening test to find out the chance of your baby having a genetic condition?

◯ Yes
◯ No
◯ Don't know

Which of these bests describe why you would have a screening test? (choose up to 2)

- ☐ So I can prepare for the possibility of having a child with a genetic condition.
- ☐ So I can prepare for the possibility of having a child with a disability.
- ☐ To help me make a decision about whether or not to continue with the pregnancy.
- ☐ I would want as much information about the baby as possible.
- ☐ My partner or family would want me to.
- ☐ My friends have had screening in their pregnancies.
- ☐ It is part of the antenatal care service.

Which of these bests describe why you would not have a screening test? (choose up to 2)

- ☐ I would never terminate an affected pregnancy so there would be no point taking the screening test.
- ☐ It would cause a lot of anxiety if I found out there was a high chance of having a baby with a genetic condition.
- ☐ I wouldn't want to have to make a decision about what to do next.
- ☐ My partner or family would not want me to.
- ☐ It would cause a lot of anxiety if I found out there was a high chance of having a baby with a disability.
- ☐ It doesn't give me a certain result.
- ☐ I would prefer not to know.

Survey Progress:

Please read the following information about diagnostic tests in pregnancy. Diagnostic tests in pregnancy can tell you for certain if your baby has a genetic condition. There are 2 types of diagnostic test:

Chorionic villus sampling (CVS) involves removing and testing a small sample of cells from the placenta.

Amniocentesis involves removing and testing a small sample of cells from the fluid around the baby (amniotic fluid).

For these tests, a needle is put through the mother's belly to collect the cells from the placenta or amniotic fluid.

Around 1 in every 200 women (0.5%) who has a diagnostic test will miscarry as a result of the test.

Now please answer the following questions.

For me, diagnostic tests in pregnancy are

◯ 1 - Harmful
◯ 2
◯ 3
◯ 4
◯ 5 - Beneficial

For me, diagnostic tests in pregnancy are

◯ 1 - Unimportant
◯ 2
◯ 3
◯ 4
◯ 5 - Important

For me, diagnostic tests in pregnancy are

◯ 1 - A bad thing
◯ 2
◯ 3
◯ 4
◯ 5 - A good thing

For me, diagnostic tests in pregnancy are

◯ 1 - Unhelpful
◯ 2
◯ 3
◯ 4
◯ 5 - Helpful

Would you have a diagnostic test to find out the chance of your baby having a genetic condition?

◯ Yes
◯ No
◯ Don't know

Which of these bests describe why you would have a diagnostic test? (choose up to 2)

☐ So I can prepare for the possibility of having a child with a genetic condition.
☐ So I can prepare for the possibility of having a child with a disability.
☐ To help me make a decision about whether or not to continue with the pregnancy.
☐ I would want as much information about the baby as possible.
☐ My partner or family would want me to.
☐ My friends have had diagnostic testing in their pregnancies.
☐ It is part of the antenatal care service.

Which of these bests describe why you would not have a diagnostic test? (choose up to 2)

☐ Because of the risk of miscarriage.
☐ Because the procedure might be painful or uncomfortable.
☐ I would never terminate an affected pregnancy so there would be no point taking the test.
☐ It would cause a lot of anxiety if the baby was found to be affected.
☐ I wouldn't want to have to make a decision about whether to terminate the pregnancy.
☐ My partner or family wouldn't want me to.
☐ I would prefer not to know.

Survey Progress:

If you were deciding whether or not to have a prenatal test, which of these would affect your decision? (choose up to 4)

☐ Whether I felt I could cope raising a child with a genetic condition.
☐ Whether I felt I could cope raising a child with a disability.
☐ My religious beliefs.
☐ Wanting to have as much information as possible about the baby.
☐ My personal views on termination of pregnancy.
☐ The impact of a child with a genetic condition on my family.
☐ Not wanting to interfere with nature.
☐ My family's religious beliefs.
☐ Not wanting to risk the safety of my baby.
☐ The advice of healthcare professionals.
☐ My family's views on termination of pregnancy.

To help you make a decision about whether or not to have a prenatal test, where would you go for information?

	Strongly agree	Agree	Neutral	Disagree	Strongly disagree
Healthcare professional	◯	◯	◯	◯	◯
Friends and family	◯	◯	◯	◯	◯
Internet	◯	◯	◯	◯	◯
Religious leader	◯	◯	◯	◯	◯
Parent support organisation	◯	◯	◯	◯	◯

If you had a question about prenatal testing, how important would it be to speak to someone from the same ethnic group as you?

◯ Very important
◯ Important
◯ Neutral
◯ Unimportant
◯ Very unimportant

Which of these support organisations have you heard of? (choose as many as needed)

☐ Tommy's Midwives Helpline for Black and Black Mixed-Heritage women
☐ Antenatal Results and Choices (ARC)
☐ Five x More
☐ None of these

Survey Progress:

Please answer these next questions, thinking about your own personal feelings towards the healthcare system.

Healthcare professionals sometimes hide information from patients who belong to my ethnic group.

◯ Strongly agree
◯ Agree
◯ Neither agree nor disagree
◯ Disagree
◯ Strongly disagree

Healthcare professionals have the best interests of people of my ethnic group in mind.

◯ Strongly agree
◯ Agree
◯ Neither agree nor disagree
◯ Disagree
◯ Strongly disagree

People of my ethnic group should not confide in healthcare professionals because it will be used against them.

◯ Strongly agree
◯ Agree
◯ Neither agree nor disagree
◯ Disagree
◯ Strongly disagree

People of my ethnic group should be suspicious of information from healthcare professionals.

◯ Strongly agree
◯ Agree
◯ Neither agree nor disagree
◯ Disagree
◯ Strongly disagree

People of my ethnic group cannot trust healthcare professionals.

◯ Strongly agree
◯ Agree
◯ Neither agree nor disagree
◯ Disagree
◯ Strongly disagree

People of my ethnic group should be suspicious of modern medicine.

◯ Strongly agree
◯ Agree
◯ Neither agree nor disagree
◯ Disagree
◯ Strongly disagree

Healthcare professionals treat people of my ethnic group like "guinea pigs".

◯ Strongly agree
◯ Agree
◯ Neither agree nor disagree
◯ Disagree
◯ Strongly disagree

People of my ethnic group receive the same medical care from healthcare professionals as people from other groups.

◯ Strongly agree
◯ Agree
◯ Neither agree nor disagree
◯ Disagree
◯ Strongly disagree

Healthcare professionals do not take the medical complaints of people of my ethnic group seriously.

◯ Strongly agree
◯ Agree
◯ Neither agree nor disagree
◯ Disagree
◯ Strongly disagree

People of my ethnic group are treated the same as people of other groups by healthcare professionals.

◯ Strongly agree
◯ Agree
◯ Neither agree nor disagree
◯ Disagree
◯ Strongly disagree

In most hospitals, people from all ethnic groups receive the same kind of care.

◯ Strongly agree
◯ Agree
◯ Neither agree nor disagree
◯ Disagree
◯ Strongly disagree

I have personally been poorly or unfairly by healthcare professionals because of my ethnicity.

◯ Strongly agree
◯ Agree
◯ Neither agree nor disagree
◯ Disagree
◯ Strongly disagree

Survey Progress:

Part 2: Understanding of genetics and the different tests available in pregnancy

In this section, you'll be asked about your understanding of genetics.

Please don't worry if you don't know the answers!

This is not a test.

Whatever answers you give will help us find out what information people understand, and what information people find more difficult.

I have heard of the word...

	Yes	No	Don't know
Gene	◯	◯	◯
Chromosome	◯	◯	◯
DNA	◯	◯	◯
Genome	◯	◯	◯

I know the meaning of the word...

	Yes	No	Don't know
Gene	◯	◯	◯
Chromosome	◯	◯	◯
DNA	◯	◯	◯
Genome	◯	◯	◯

Survey Progress:

In the following sections, you'll read some scenarios and be asked about the different tests available during pregnancy.

The scenarios are included to help frame the questions. Please try to answer the questions based on what you know.

Remember - this is not a test.

Please don't worry if you don't know the answers!

The combined screening test

Read the scenario and then answer the questions below.

The combined screening test combines a blood test taken from the mother with an ultrasound scan. It is available between weeks 10 and 14 of pregnancy.

Stella is aged 27 and is 12 weeks pregnant with her second child. She is offered the combined screening test.

The combined screening test will tell Stella about the chance of her baby having Down syndrome, Edwards' syndrome and Patau's syndrome.

◯ True ◯ False ◯ Don't know

Stella received a low chance result from the combined screening test in her last pregnancy, so the result will be the same in this pregnancy.

◯ True ◯ False ◯ Don't know

The combined screening test will test Stella's baby for autism.

◯ True ◯ False ◯ Don't know

The combined screening test cannot harm Stella or her baby.

◯ True ◯ False ◯ Don't know

Stella's baby won't have Down syndrome because Down syndrome only happens in pregnancies of women over 35.

◯ True ◯ False ◯ Don't know

Survey Progress:

The quadruple blood screening test

Read the scenario and then answer the questions below.

If it hasn't been possible to get the measurements needed from your scan, or you're more than 14 weeks into your pregnancy, you'll be offered a test called the quadruple blood screening test.

In the quadruple blood screening test, a blood sample is taken from the mother. It is available between weeks 14 and 20 of pregnancy.

Jade is 16 weeks pregnant and is offered the quadruple test.

The quadruple test is less accurate than the combined screening test.

○ True ○ False ○ Don't know

The quadruple test is used to test for Down syndrome, Edwards' syndrome and Patau's syndrome.

○ True ○ False ○ Don't know

If Jade gets a high chance result from the quadruple test, it means that her baby has a genetic condition for certain.

○ True ○ False ○ Don't know

Jade can find out the sex of her baby from the quadruple test.

○ True ○ False ○ Don't know

Survey Progress:

Non-invasive prenatal testing (NIPT)

Read the scenario and then answer the questions below.

Non-invasive prenatal testing (NIPT) involves taking a blood sample from the mother.

It can provide a more accurate result than the combined screening or quadruple test. It's offered if the result you get from either of these tests is high chance (higher than 1 in 150).

Zoe is 13 weeks pregnant. Results from her screening test showed a high chance result for Edwards' syndrome. Zoe and her partner Frank decide to have further testing using NIPT.

NIPT comes with a risk of complications for Zoe and the baby.

◯ True ◯ False ◯ Don't know

NIPT is offered in all pregnancies.

◯ True ◯ False ◯ Don't know

NIPT will tell Zoe and Frank for certain whether their baby has Edwards' syndrome.

◯ True ◯ False ◯ Don't know

NIPT screens for all genetic conditions.

◯ True ◯ False ◯ Don't know

If Zoe and Frank receive a high chance result from NIPT, they'll need further invasive testing to find out for certain if their baby has Edwards' syndrome.

◯ True ◯ False ◯ Don't know

Survey Progress:

Amniocentesis

Read the scenario and then answer the questions below.

Amniocentesis is offered if blood or scan results suggest your baby could have a genetic condition.

Amniocentesis involves removing and testing a small sample of cells from the fluid around the baby (amniotic fluid).

Lauren is 15 weeks pregnant and has been offered amniocentesis after receiving a high chance result for Patau's syndrome.

Amniocentesis will tell Lauren for certain whether her baby has Patau's syndrome.

◯ True ◯ False ◯ Don't know

Amniocentesis is completely safe to Lauren and her baby.

◯ True ◯ False ◯ Don't know

Amniocentesis can be performed as early as 10 weeks in pregnancy.

◯ True ◯ False ◯ Don't know

Survey Progress:

Sickle cell - part 1

Read the scenario and then answer the questions below.

Sickle cell is a blood condition that causes the body to make unusually shaped red blood cells. These blood cells can cause problems because they do not live as long as healthy blood cells and they can block blood vessels.

Kelly is 12 weeks pregnant with her third child. Blood tests confirm that both her and her partner John are carriers of sickle cell. This means they have the sickle cell trait.

Sickle cell is a genetic condition and is not contagious.

◯ True ◯ False ◯ Don't know

Kelly and John's baby cannot be affected by sickle cell because Kelly and John do not have sickle cell - they are only carriers of the condition.

◯ True ◯ False ◯ Don't know

Kelly and John's baby cannot be affected by sickle cell because Kelly and John's two other children do not have sickle cell.

◯ True ◯ False ◯ Don't know

The chance that Kelly and John's baby will be affected by sickle cell is:

◯ Zero chance
◯ 1 in 4 (25%)
◯ 1 in 2 (50%)
◯ 1 in 1 (100%)
◯ Don't know

Survey Progress:

Sickle cell - part 2

Read the scenario and then answer the questions below.

Melanie is 14 weeks pregnant and identifies as being of Caribbean descent. She has the sickle cell trait.

Her partner George describes himself as being of Irish descent. George believes he does not have the sickle cell trait.

It is not possible for George to have the sickle cell trait because he is of Irish descent and sickle cell only affects people of African and Caribbean descent.

◯ True ◯ False ◯ Don't know

If Melanie has sickle cell trait but George does not, their baby will not have sickle cell or the sickle cell trait.

◯ True ◯ False ◯ Don't know

Melanie's sickle cell trait will eventually develop into sickle cell.

◯ True ◯ False ◯ Don't know
